# Investigation of the inhibitory properties of azo-dyes on chorismate synthase from *Paracoccidioides brasiliensis*

**DOI:** 10.1080/14756366.2024.2427175

**Published:** 2024-12-10

**Authors:** Katharina Fuchs, Massimo G. Totaro, Marina Toplak, Aleksandar Bijelic, Peter Macheroux

**Affiliations:** Institute of Biochemistry, Graz University of Technology, Graz, Austria

**Keywords:** Azo-dyes, computational chemistry, drug design, flavoenzymes, inhibitor

## Abstract

The efficient inhibition of 5-enolpyruvylshikimate-3-phosphate synthase (EPSPS) by the broad-spectrum herbicide glyphosate validates the shikimate pathway as a promising target for developing antimicrobial, fungicidal and herbicidal agents. The last enzyme of this pathway, chorismate synthase (CS), catalyses an unusual reaction, making it an attractive target for novel inhibitors. Therefore, we tested a series of azo-dyes for their inhibitory potential against CS from the pathogenic fungus *Paracoccidioides brasiliensis* (*Pb*CS) and identified the azo-dye PH011669 that exhibits a dissociation (*K*_d_) and 50% inhibitory constant (IC_50_) of 1.1 ± 0.1 and 10 ± 1 µM, respectively. Molecular docking and MD simulations provided insight into the mode of inhibition, showing that PH011669 binds to the enzyme’s active site primarily through electrostatic interactions. Thus, our study is the first to integrate structural and computational methods to guide future efforts towards designing the next generation of CS inhibitors.

## Introduction

**Figure 1.**
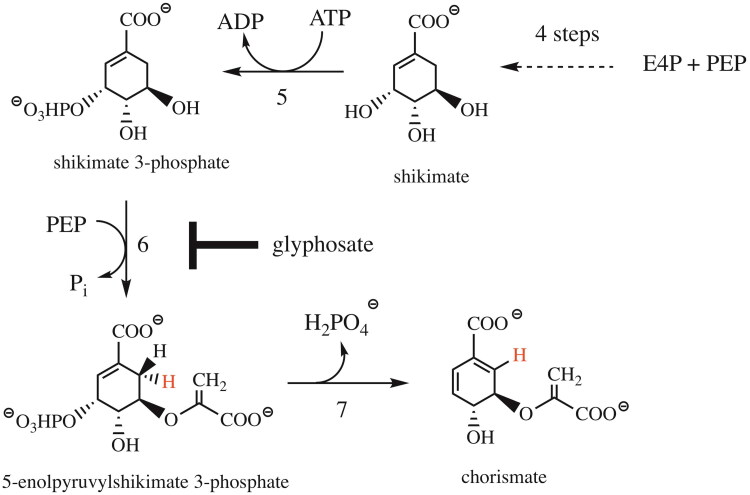
The last steps of the shikimate pathway. Erythrose 4-phosphate (E4P) and phosphoenolpyruvate (PEP) are used as building blocks to synthesise shikimate (top right) in four enzymatic steps. Shikimate is phosphorylated by shikimate kinase at the expense of ATP to shikimate-3-phosphate (step 5). In step six, 5-enolpyruvylshikimate-3-phosphate synthase (EPSPS) catalyses the conversion of shikimate-3-phosphate to 5-enolpyruvylshikimate-3-phosphate (EPSP, left). EPSPS is the target of the broad-band herbicide glyphosate. The seventh step of the common pathway is catalysed by chorismate synthase involving the stereospecific *anti*-1,4-elimination of the 3-phosphate and the 6*proR*-hydrogen (bottom).

Chorismate synthase (CS, EC 4.6.1.4) catalyses the seventh and last common step in the shikimate pathway, which is responsible for the biosynthesis of crucial aromatic compounds, such as the aromatic amino acids phenylalanine, tyrosine and tryptophan, as well as a host of other essential aromatic biomolecules (e.g. alkaloids, folates, lignin, ubiquinones, siderophores and plant secondary compounds). The enzyme-catalysed conversion of 5-enolpyruvylshikimate-3-phosphate (EPSP) to chorismate involves an anti-1,4-elimination of the phosphate and the C-(6*pro*R)-hydrogen (Figure 1).

The shikimate pathway is essential for prokaryotes, protozoa, fungi and plants. The sixth enzyme of the common pathway, EPSP synthase (EPSPS), is the target of the prominent herbicide glyphosate (Figure 1), and thus, blocking the pathway is clearly deleterious for organisms that depend on a functioning shikimate pathway for the biosynthesis of essential aromatic compounds.

At first, the absolute requirement for reduced FMN in the CS-catalysed reaction was puzzling since the 1,4-anti elimination of a phosphate group and a hydrogen atom is redox neutral and thus does not require the assistance of a redox cofactor. However, it was also realised that abstraction of the 6*pro*R-hydrogen is challenging owing to its high p*K*a value, estimated to be ≈30^1^. Detailed mechanistic studies involving substrate derivates and rapid reaction kinetics, as well as density functional calculations, have provided evidence that the reduced flavin acts as a transient electron donor, thereby facilitating the bond-breaking steps^2–5^. The crystal structure of CS from *Streptococcus pneumoniae* (*Sp*CS) in complex with (oxidised) FMN and its substrate EPSP agrees with such a mechanism and rationalises the requirement of a redox cofactor in a redox neutral elimination reaction^6^.

In view of the success of glyphosate as one of the leading herbicides worldwide, CS appeared to be a very attractive drug target^7^^,^^8^. First attempts to identify an inhibitor of CS were already initiated in the 1990s before the first crystal structure was reported by Mclean and Ali, and thus mainly relied on the structure of EPSP as a lead to synthesise substrate analogs with potential inhibitory properties^6^. As CS has a strict stereospecificity for eliminating the *proR*-hydrogen, fluorinated substrate analogs were synthesised, i.e. 6(S)- and 6(R)-6-fluoro-EPSP^9^. It was shown that 6(S)-6-fluoro-EPSP is a very slow substrate exhibiting a 200-fold slower turnover than EPSP^10^. This result suggested that the reported antimicrobial properties of 6-fluoro-shikimate are probably not due to the inhibition of CS but the inhibition of 4-aminobenzoic acid synthesis, as proposed earlier^11^. On the other hand, 6(R)-6-fluoro-EPSP lacks the (6*pro*R)-hydrogen and is therefore not a substrate of CS. This compound was found to be a competitive inhibitor of CS from *Neurospora crassa,* exhibiting a K_i_ of 3.0 ± 0.3 µM^9^. However, it is still unclear whether the reported antimicrobial activity of (6R)-6-fluoro-shikimate is due to CS inhibition.

**Figure 2.**
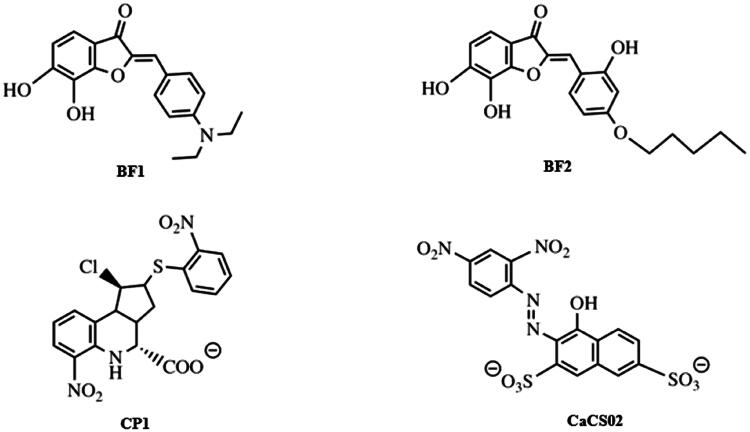
CS inhibitors reported by Thomas^12^ and Rodrigues-Vendramini^13^.Top panel: initial hit (left) and the improved inhibitor (right) reported by Thomas and colleagues^12^. Bottom panel: inhibitors found by virtual screening by Rodrigues-Vendramini and collegues^13^. IC_50_ values for compounds BF1, BF2, CP1 and CaCS02 were 8, 0.22, 47 and 29 µM, respectively.

In 2003, the use of an extensive library of diverse small molecules to screen for an inhibitor of *Sp*CS was reported^12^. Based on the initial hit, a benzofuran-3[2H]-one (BF1) derivative with a 50% inhibitory constant (IC_50_) of 8 µM, modification of the alkoxy chain length of the phenyl ring (BF2) resulted in a further improvement of the inhibitory properties, as shown in Figure 2. Unfortunately, further studies to characterise the potency and applicability of these compounds were not reported.

More recently, virtual screening using a three-dimensional structure of CS from *Paracoccidioides brasiliensis* (*Pb*CS, homology model based on the crystallographic structure of the *Saccharomyces cerevisiae* enzyme) in combination with molecular dynamics calculations led to the discovery of two inhibitors, a quinoline derivative (1S,2S,3aS,4S,9bR)-1-chloro-6-nitro-2-(2-nitrophenyl)sulfanyl-2,3,3a,4,5,9b-hexahydro-1H-cyclopenta[c]quinoline-4-carboxylic acid (CP1) and the azo dye nitrazine yellow 1-(2,4-dinitrophenylazo)-1-hydroxylnaphthalene-3,6-disulfonic acid (CaCS02) (Figure 2). The dimorphic fungus *P. brasiliensis* was chosen as a target because it causes paracoccidioidomycosis (PCM), an invasive fungal disease, which is the most prevalent systemic mycosis in Latin America. In Brazil, for example, PCM is responsible for approximately half of the deaths caused by systemic mycoses^14^^,^^15^.

Compounds CP1 and CaCS02 bind to *Pb*CS with dissociation constants of 64 ± 1 and 20 µM and inhibit the enzyme with IC_50_ values of 47 ± 5 and 29 ± 3 μM, respectively^13^. Since several azo-dyes featuring different substitution patterns at the phenyl and naphthyl ring are commercially available, we decided to extend our study by testing a series of azo-dyes for their binding affinity to *Pb*CS, leading to the discovery of an azo-dye (PH011669) with a ca. 20-fold higher affinity than the initial hit and an IC_50_ value of 10 µM. To gain further insight into the binding of PH011669 to *Pb*CS, we conducted molecular docking and molecular dynamics (MD) simulations.

## Materials and methods

### Azo-dyes tested in the current study

Supplementary Table 1 provides the names and structures of the azo-dyes investigated in the current study. The compounds were purchased from Sigma-Aldrich and used without further purification.

### Production and purification of CSs

CSs from 16 organisms were produced. The genes coding for the respective proteins were cloned and protein production was performed in different pET *E. coli* T7 expression vectors and *E. coli* strains (Supplementary Table 2). The pET-21a(+) plasmid containing the gene coding for *Pb*CS was received from Dr. Flavio AV Seixas (State University of Maringá, Brazil). Recombinant protein production and purification from all CSs, except for CS from *Mycobacterium tuberculosis*, which was produced and purified as reported by Dias and colleagues^16^, was adapted from Rodrigues-Vendramini and coworkers^13^. Briefly, *E. coli* was grown in an LB medium containing the appropriate antibiotic and inoculated with a fresh overnight culture to an optical density at 600 nm (OD_600_) of 0.1. The main cultures were grown to an OD_600_ of 0.7 at 37 °C and 140 rpm. To start protein production, isopropyl-α-D-1-thiogalactopyranoside (IPTG) was added to a final concentration of 0.1 mM. After induction, the main cultures were incubated at 20 °C and 140 rpm overnight (∼16 h). The cells were then harvested by centrifugation (5,500 × g for 15 min), and the resulting cell pellet was stored at −20 °C until further use. To obtain pure protein, cell pellets were resuspended in lysis buffer (50 mM NaH_2_PO_4_, 150 mM NaCl, 10 mM imidazole, pH 8) and lyzed by sonication (2 × 10 min) using a Labsonic P sonication probe (B. Braun Biotech, Berlin, Germany). After sonication, the lysate was centrifuged (38,500 × g for 45 min) and loaded onto a 5 mL nickel-nitrilotriacetic acid (Ni-NTA) column. The column was equilibrated with lysis buffer, and the target protein was eluted with elution buffer (50 mM NaH_2_PO_4_, 150 mM NaCl, 300 mM imidazole, pH 8). Fractions containing the target protein were pooled and dialysed against 50 mM NaH_2_PO_4_ and 150 mM NaCl (pH 8) overnight. The target protein was then concentrated using an ultrafiltration device (30-kDa cut-off, Merck-Millipore, Darmstadt, Germany). The purified protein was flash-frozen using liquid nitrogen and stored at −80 °C until further use.

### Spectrophotometric titrations/binding assay

For the binding studies, PH011669 was dissolved in 50 mM NaH_2_PO_4_ and 150 mM NaCl at pH 8, diluted to a final concentration of approximately 10 µM (A_580nm_ ∼0.25; 800 µL), and transferred to a quartz cuvette (with 800 µL of buffer in the reference cuvette). After recording an initial UV-visible absorption spectrum between 300 and 800 nm, 10 µL aliquots of *Pb*CS (∼55 µM) were added, and spectral changes were monitored after each addition. To quantify the binding affinity of PH011669 to *Pb*CS, we plotted the relative absorption changes at 510 nm as a function of the protein concentration in the cuvette^17^.

### Isothermal titration calorimetry (ITC)

Isothermal titration calorimetry (ITC) was performed on a MicroCal PEAQ-ITC. All ITC experiments were conducted in storage buffer (50 mM NaH_2_PO_4_, 150 mM NaCl, pH 8) at 25 °C and a stirring rate of 750 rpm. Titration experiments consisted of 13 injections (first injection: 0.4 µL; duration time: 0.8 s; spacing time: 300 s; 2nd–13th injection: 3 µL; duration time: 6 s; spacing time: 150 s) and were performed in triplicates. The azo-dye PH011669 (500 µM) was loaded into the injection syringe and titrated into the sample cell containing *Pb*CS (25 µM); the reference cell was filled with degassed ddH_2_O. Dissociation constants were obtained with the MicroCal PEAQ-ITC Analysis Software (Malvern) using the “one set of sites fitting” model.

### Inhibition assay

The influence of PH011669 on the activity of *Pb*CS was tested by forward coupling the CS reaction with the anthranilate synthase reaction. The final reaction solution contained 80 μM EPSP, 4 μM *Pb*CS, 7 µM YcnD, 20 μM anthranilate synthase, 4 mM MgSO4, 10 mM L-glutamine (L-Gln), 30 mM (NH_4_)_2_SO_4_, 1 mM dithiothreitol (DTT) and different concentrations of the inhibitor (0.1–250 µM). The assay was prepared in 10 mM potassium phosphate at pH 7.6 and pipetted into a 96-well plate. The reaction was started by the addition of 500 µM NADPH. *Pb*CS activity could be studied spectrofluorometrically at 37 °C by following the increase in the fluorescence intensity at 390 nm (λ_ex_ = 340 nm) for 5 min, which correlates with the amount of anthranilate formed in the course of the reaction. The slopes of the initial velocities were determined and plotted as a function of the log of the corresponding PH011669 concentration (triplicates were determined).

### Structure prediction of PbCS

The structure of *Pb*CS was modelled using AlphaFold2 via the Collaboratory service from Google Research (https://colab.research.google.com/github/sokrypton/ColabFold/blob/main/beta/AlphaFold2_advanced.ipynb)^18^. Briefly, the sequence of *Pb*CS was submitted to AlphaFold2, and, as a first step, a multiple sequence alignment was performed employing the mmseq2 method^19^. Five models per enzyme were predicted and ranked by pLDDT (predicted local distance difference test). The five predicted *Pb*CS models were superimposed on the crystal structure of *Sp*CS complexed with oxidised FMN and EPSP (PDB ID: 1QXO^6^). The model with the lowest Cα-RMSD to PDB entry 1QXO and the highest pLDDT score was refined with the Amber-Relax protocol of the AlphaFold2 pipeline and validated by the MOLPROBITY and RAMPAGE servers^20^. *Pb*CS was modelled as a homotetramer (biological unit), but only one protomer was used for the subsequent docking experiment and MD simulations.

### Molecular docking

Molecular docking was performed with AutoDock Vina (version 1.2.1)^21^ to study the binding mode of PH011669 in the active site of *Pb*CS and provide initial coordinates and topology parameters for the MD simulations. The predicted AlphaFold model was used as the receptor, and an FMN molecule was added to the model before docking by superimposing the catalytic core of the crystal structure 1QXO onto our model. The structure of PH011669 was generated with the NCI Online SMILES Translator and Structure File Generator (https://cactus.nci.nih.gov/translate/). The model structures of PH011669 and *Pb*CS-FMN were then formatted into pdbqt files using AutoDockTools (version 1.5.7)^22^, specifying and sampling all rotatable bonds and computing partial charges for both structures. Binding poses of PH011669 were searched in a grid box of 14 × 16 × 10 Å^3^ (spacing = 1.0 Å) centred on the substrate EPSP after superimposing the AlphaFold model of *Pb*CS on the crystal structure 1QXO. Docking exhaustiveness was set to 50. AutoDock Vina was run 10 times to verify the consistency and reliability of the docking results. The final docking settings were validated by re-docking EPSP into the active site of 1QXO, which lacked the substrate EPSP. The resulting docking poses closely resembled the EPSP pose found in the crystal structure, verifying our docking protocol. Upon docking, the binding poses of PH011669 were compared to that of EPSP in 1QXO and evaluated by docking scores and for reasonability. Poses that deviated significantly from the pose of EPSP in the reference structure 1QXO were excluded. The pose with the most reasonable orientation and highest docking score was selected as input for the following MD simulations.

### Molecular dynamics

The MD experiment was performed using GROMACS 2022^23^, as previously described^24^. The receptor (i.e. *Pb*CS-FMN) and ligand (i.e. PH011669) GRO coordinates and topology files were generated and merged to obtain the complex (i.e. *Pb*CS-FMN-PH011669) GRO and topology files. The flavin cofactor was parameterised in its reduced form (i.e. FMNH_2_), as the enzyme requires reduced FMN for activity.

Three systems were investigated by MD: apo*Pb*CS, *Pb*CS-PH011669 (binary system) and *Pb*CS-FMN-PH011669 (ternary system). The MD unit cell was defined as a dodecahedron with a minimum distance of 1.0 nm between the complex and the box edges. The system was solvated with the SPC/E water model and neutralised by adding Na^+^ ions. The system was then subjected to standard energy minimization and equilibration. Energy minimization was performed using the steepest descent algorithm with a maximum force of 1000 kJ/mol/nm and a maximum of 5000 steps. The equilibration step consisted of two phases: NVT (constant number of particles, volume and temperature) and NPT (constant number of particles, pressure and temperature). The NVT phase was run for 100 ps with a temperature coupling of 300 K using the v-rescale thermostat. The NPT phase was run for 200 ps with a pressure coupling of 1 bar using the Parrinello-Rahman barostat.

Initially, three independent MD production runs were performed for each system starting from the equilibration output, each for 300 ns with initial velocity generation (i.e. 900 ns for each system). For the binary system, we needed to increase the sampling and thus performed six more independent runs of 300 ns. The parameters for the production runs were the same for all systems: temperature and pressure were set to 300 K and 1 bar by the v-rescale thermostat and Parrinello-Rahman barostat, respectively; bonds were constrained using the LINCS algorithm; the Verlet cutoff scheme was used to process intra-atomic interactions; the PME method was implemented to account for Coulombic and Lennard-Jones interactions; and a van der Waals cutoff radius of 1.0 was applied.

Note that only one protomer was used for all calculations to reduce computational cost.

### Molecular dynamics analysis

The analysis density module of the MDAnalysis python toolkit^25^^,^^26^ was applied to calculate residence densities for PH011669 from the trajectories of the binary and ternary systems using default parameters. The resulting density maps were visualised as volumes (carve = 1.6 Å) using PyMOL^27^.

A principal component analysis (PCA) of the PH011669 across the binary and ternary complex trajectories was performed. First, all the trajectories were aligned on the receptor backbone; then, the PH011669 coordinates were extracted into separate sub-trajectories. The full ensemble of these sub-trajectories was used to train the PCA algorithm provided by the MDAnalysis.analysis package, which was then used to transform the individual ones. Finally, the first two principal components were used to generate the data for the kernel density estimate (KDE) plots.

**Figure 3.**
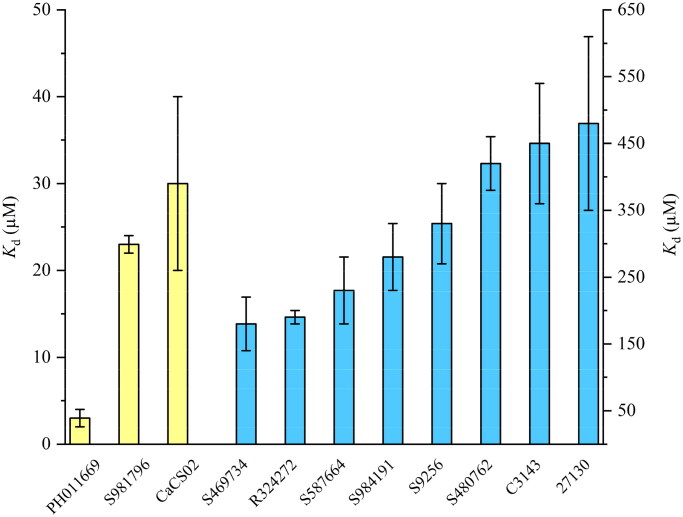
Azo-compounds PH011669, S981796, CaCS02, S469734, R324272, S587664, S984191, S9256, S480762, C3143 and 27130 ranked according to their *K*_d_-values against *Pb*CS. To quantify the binding affinities of the azo-compounds to *Pb*CS, the relative absorption changes at a certain wavelength were plotted as a function of the protein concentration in the cuvette, with the standard deviations displayed as error bars (from three titrations). The scales for the yellow and blue bars are indicated on the left and right sides, respectively.

### Binding free energy calculations

Molecular mechanics/Poisson–Boltzmann surface area (MM/PBSA) calculations were performed with the program gmx_MMPBSA^28^ to approximate binding energies for the enzyme-inhibitor complexes. The last 30 ns of each production run (i.e. 3 × 30 ns for the ternary and 9 × 30 ns for the binary system) were used for energy calculations. The internal dielectric constant of the solute, the internal dielectric constant of the gas phase, temperature, salt concentration and Generalised Born method value were set to 1, 80, 298.15 K, 0.15 M and 5, respectively. Per-residue energy decomposition analysis was performed using default parameters to determine the binding energy contribution of all residues within 6 Å of PH011669. The obtained data were averaged over three (ternary system) and nine (binary system) independent trajectories.

### ADME prediction

The absorption, distribution, metabolism and excretion (ADME) properties for PH011669 and an optimised version of PH011669 were assessed using the SwissADME web tool^29^. For this purpose, the SMILES string for PH011669 and its optimised version were submitted to the server.

## Results and discussion

### Azo-dyes as inhibitors of chorismate synthases

As the azo-dye CaCS02 (Figure 2) showed a promising dissociation constant (20 µM) and IC_50_ value (29 µM), we screened a series of structurally similar and commercially available azo-dyes for their binding affinity using *Pb*CS. The binding affinities of azo-dyes listed in Table 1 were determined by spectrophotometric titrations employing *Pb*CS as the target enzyme. As shown in Figure 3, binding affinities varied over a wide range, with PH011669 exhibiting by far the lowest dissociation constant of 1.1 ± 0.1 µM.

**Figure 4.**
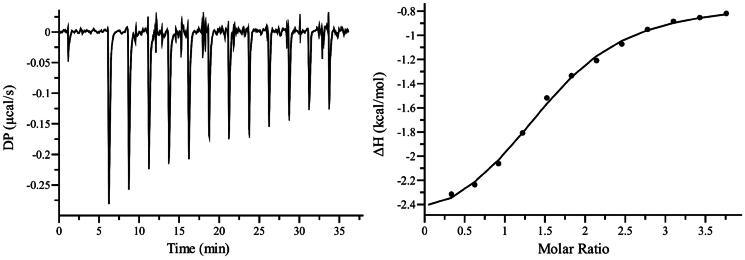
Determination of the dissociation constant of PH011669 to *Pb*CS by isothermal titration calorimetry. The experiment was performed in triplicates, and data were fitted with a single site binding model.

**Table 1.** Affinity of PH011669 towards CSs from different organisms.

Species	Classification	Yield^a^	*K*_d_ (µM)
*Anabaena variabilis*	cyanobacterium	+++	1.8 ± 0.1
*Aquifex aeolicus*	Gram^−^ bacterium	+++	3.2 ± 0.1
*Botrytis fuckeliana*	fungus	+++	4.0 ± 0.6
*Candida albicans*	fungus	++	4.6 ± 0.7
*Corydalis sempervirens*	plant	+	1.0 ± 0.1
*Escherichia coli*	Gram^−^ bacterium	++	3.7 ± 1.0
*Methanobacterium thermoautotrophicum*	Gram^+^ bacterium	+	2.7 ± 0.0
*Mycobacterium tuberculosis*	Gram^+/−^ bacterium	n. s.	–
*Neurospora crassa*	fungus	+	3.5 ± 0.1
*Paracoccidioides brasiliensis*	fungus	+++	1.1 ± 0.0
*Saccharomyces cerevisiae*	yeast	+++	2.4 ± 0.1
*Solanum lycopersicum*	plant	++	1.8 ± 0.1
*Staphylococcus aureus*	Gram^+^ bacterium	+	3.7 ± 0.1
*Tetrahymena thermophila*	unicell. eukaryote	+	4.4 ± 0.1
*Thermotoga marítima*	Gram^−^ bacterium	+	1.5 ± 0.0
*Toxoplasmodium gondii*	protozoon	+	1.7 ± 0.0

^a^Yields of purified protein are (+) = 1–4.9 mg/g cell wet weight, (++) = 5–9.9 mg/g cell wet weight and (+++) > 10 mg/g cell wet weight.

n. s.: not soluble; K_d_: dissociation constant of PH011669.

**Table 2.** Docking scores, predicted K_i_ values and number of polar interactions.

Ligand	Docking score [kcal/mol]	K_i_ [nM]	No. of polar interactions^a^
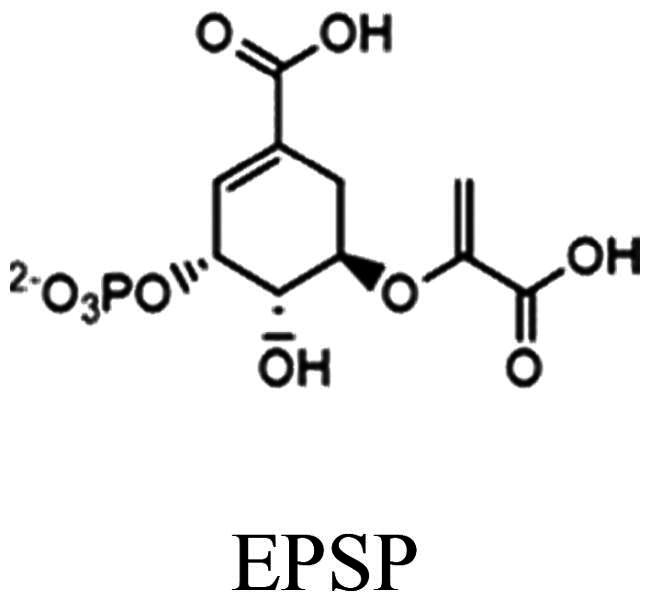	−8.4 ± 0.1	651 ± 188	9
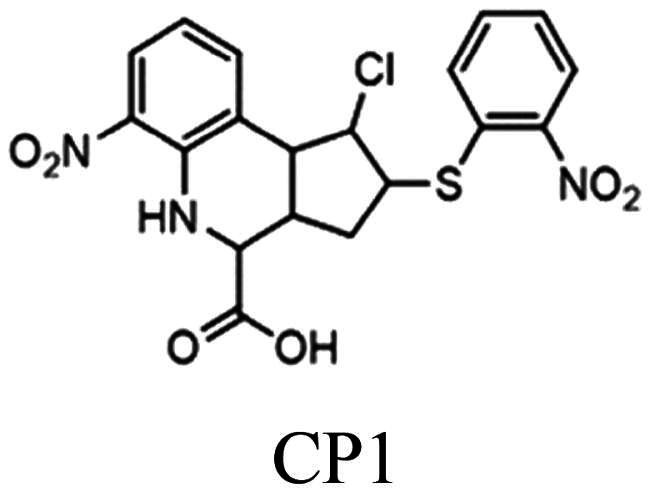	−7.9 ± 0.6	587 ± 123	5
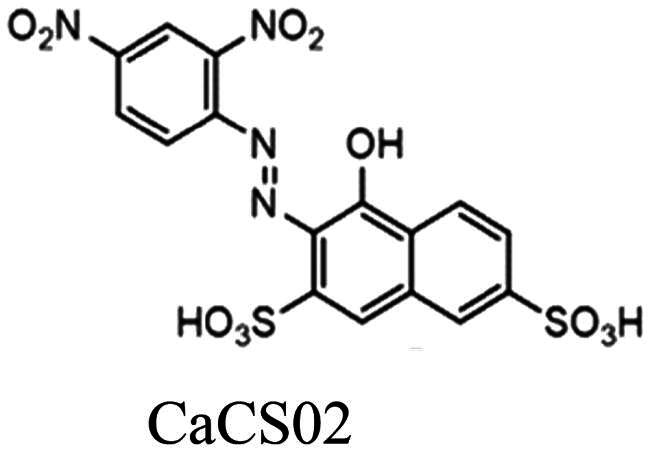	−9.8 ± 0.2	61 ± 19	8
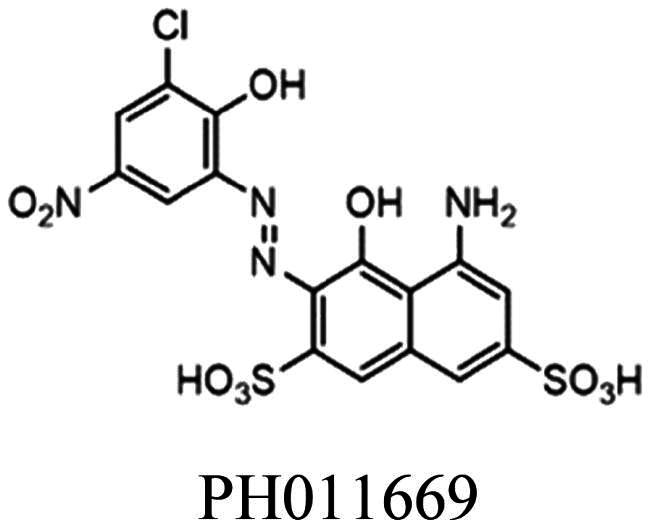	−10.2 ± 0.2	37 ± 9	9

Docking scores and K_i_-values were calculated with AutoDock Vina and represent the average of 10 independent runs.

^a^Number of polar interactions between the ligand and the enzyme in the best docking pose (i.e. best docking score).

In addition to the spectrophotometric titrations, we also determined the binding affinity of PH011669 using ITC. As shown in Figure 4, the binding of PH011669 to PbCS was an exothermic process. The experimental data were fitted with a single site binding model and yielded a dissociation constant of 3.8 ± 1.8 μM, which is comparable to that obtained by spectrophotometric titration (1.1 ± 0.1 μM).

**Figure 5.**
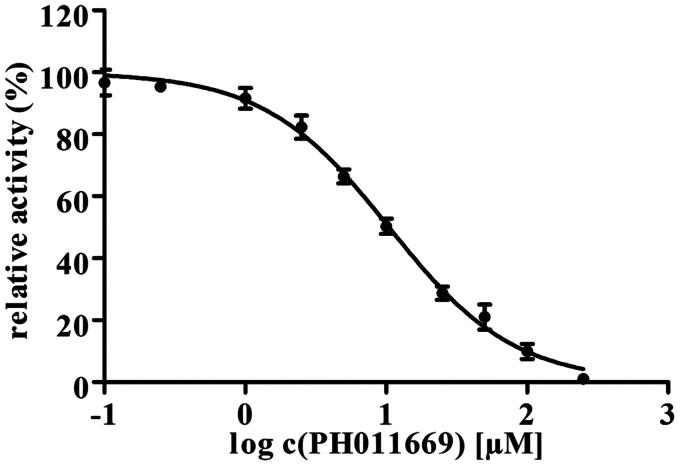
The inhibitory effect of PH011669 on the catalytic activity of chorismate synthase from *P. brasiliensis*. The assay was performed in triplicate, with the standard deviation shown as error bars; where no error bars are shown, the statistical error is smaller than the dot.

To gain insight into the affinity of PH011669 to CSs from other sources, we successfully produced fifteen CSs from bacterial, fungal and plant sources. As shown in Table 1, the highest and lowest affinity was found with the CS from the plant *Corydalis sempervirens* (1.0 ± 0.1 µM) and the fungus *Candida albicans* (4.6 ± 0.7 µM), respectively. Thus, PH011669 possesses low micromolar dissociation constants across the CSs used in our study. This indicates that the interactions governing the binding of PH011669 are fairly conserved in the active site of CSs.

### Inhibition assay

To test the inhibitory effect of PH011669 on the activity of CS, we used a coupled assay involving EPSP, CS, YcnD and anthranilate synthase. Our inhibition assay has been used to determine the ability of a drug to reduce the activity of the enzyme. The resulting IC_50_ value gives the concentration of a drug required for 50% inhibition of the enzymatic activity. Using a nonlinear curve fit based on the Hill function, an IC_50_ value of 10 ± 1 µM was determined (Figure 5). A comparable compound tested with *Pb*CS provided an IC_50_ value of 29 ± 3 µM, indicating an improved inhibitory activity^17^. Compared to the existing value and combined with the data obtained, PH011669 is an excellent *Pb*CS binder and a strong inhibitor.

**Figure 6.**
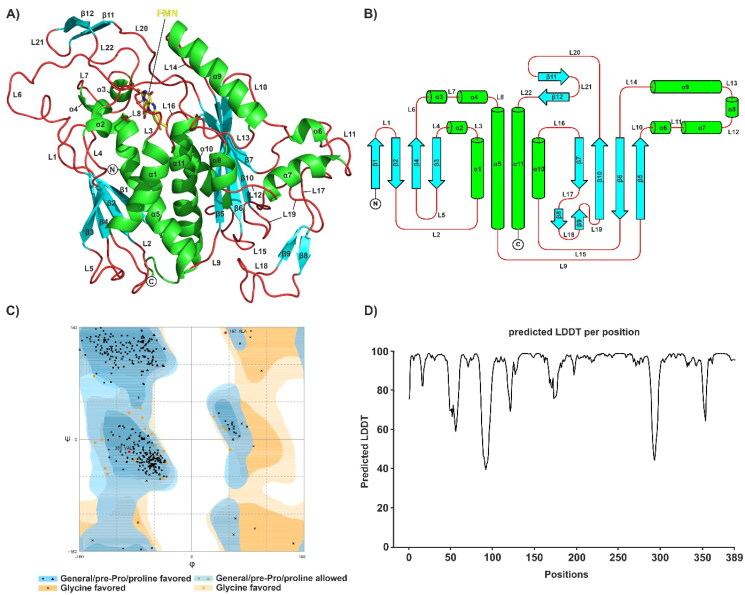
AlphaFold prediction and evaluation. (A) AlphaFold2 model of *Pb*CS colour-coded by secondary structure (α-helices, green; β-sheets, cyan; loops, red). FMN is shown as yellow sticks; secondary structure elements are annotated. (B) Topology plot of *Pb*CS colour-coded according to (A). (C) Ramachandran plot of *Pb*CS. Of all residues, 94.0%, 5.4% and 0.6% are in the favoured, allowed and outlier regions, respectively. Red squares indicate residues (A197 and V360) in the outlier region of the plot. (D) The predicted-LDDT-per-residue plot for the AlphaFold2 model of *Pb*CS.

### Evaluation of the predicted PbCS model

As no crystal structure for *Pb*CS is available, we generated an AlphaFold model. The final model exhibited an excellent average pLDDT value of 91.9% and good stereochemical quality, with 94.0% of the residues being in the favoured region of the Ramachandran plot (Figure 6 ). The final *Pb*CS model represents the active conformation of the enzymes, as the active site loops were modelled in their closed conformation. The predicted structure comprises 11 α-helices and 12 β-sheets, with its core adopting the β-α-β sandwich fold, which is unique to CSs^6^. An FMN molecule was incorporated by superimposing the binding site of our model on that of the crystal structure of the ternary complex *Sp*CS-FMN-EPSP (PDB ID: 1QXO). The calculated Cα-RMSD between our model and the experimentally determined crystal structure 1QXO is 1.25 Å, further corroborating the accuracy of our model. As AlphaFold models fail to account for conformational flexibility upon ligand binding, it was important that our model resembles the ligand-bound structure 1QXO as much as possible.

**Figure 7.**
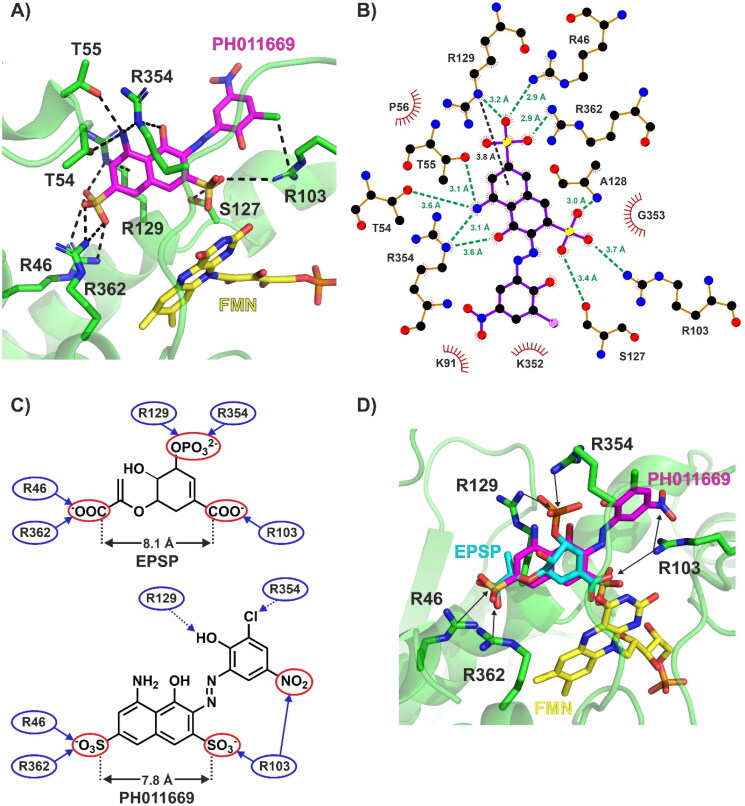
Interactions between PH011669 and *Pb*CS-FMN, as predicted by molecular docking. (A) Best binding pose of PH011669 within the active site of *Pb*CS-FMN. The enzyme is illustrated as a transparent green cartoon, with interacting residues shown as sticks. Black dashed lines indicate electrostatic and polar interactions. The interaction between R129 and the inhibitor is a cation-π interaction. (B) Protein–ligand interaction plot (LigPlot) of the calculated *Pb*CS-FMN-PH011669 complex. Green dashed lines indicate electrostatic and H-bond interactions while the black dashed line indicates cation-π interactions; bond distances are also shown. (C) Schematic comparison of the binding mode of EPSP and PH011669 in *Pb*CS. Only the main contributors to the binding of EPSP and PH011669 are depicted for clarity. Solid and dashed blue arrows indicate strong (interaction distance: <3.2 Å) and weak (interaction distance: 3.3–4.0 Å) interactions, respectively. (D) Superimposition of the best docking pose of PH011669 on the EPSP pose found in the crystal structure 1QXO. The enzyme is shown as a green cartoon, with interacting amino acids depicted as sticks. PH011669 and EPSP are illustrated as magenta and cyan sticks, respectively. Black arrows indicate interactions between the ligands and *Pb*CS.

### Molecular docking

To gain more information on the interactions between PH011669 and *Pb*CS, we performed molecular docking using the AlphaFold2-generated *Pb*CS model, including the cofactor FMN. In addition to PH011669, the substrate EPSP and the previously identified CS inhibitors CP1 and CaCS02 were docked into the active site of *Pb*CS-FMN for comparison^13^^,^^17^. PH011669 exhibited the best docking score and predicted inhibition constant (*K*_i_) among all tested ligands, outperforming the natural substrate EPSP, although both compounds are involved in the same number of polar interactions (Table 2). This is corroborated by a direct comparison of the binding affinity of EPSP and PH011669 to the CS from *N. crassa*, exhibiting *K*_d_ values of 17 µM^30^ and 3.5 µM, respectively.

As the catalytic pocket of *Pb*CS is positively charged, PH011669 binding to *Pb*CS is governed by ion-ion interactions between the inhibitor’s negatively charged sulfonate groups and the enzymes’ positively charged arginine residues (Figure 7A). The sulfonate groups of PH011669 interact with R46, R362 and R103, exhibiting bond distances between 2.9 and 3.7 Å (Figure 7B). Besides these electrostatic interactions, PH011669 is involved in a strong H-bond with the backbone amine of A128 (3.0 Å) and weaker ones with T54 (3.6 Å), T55 (3.1 Å), S127 (3.4 Å) and R354 (3.1 and 3.6 Å), which contribute only little to the binding of the inhibitor (Figure 7B).

**Figure 8.**
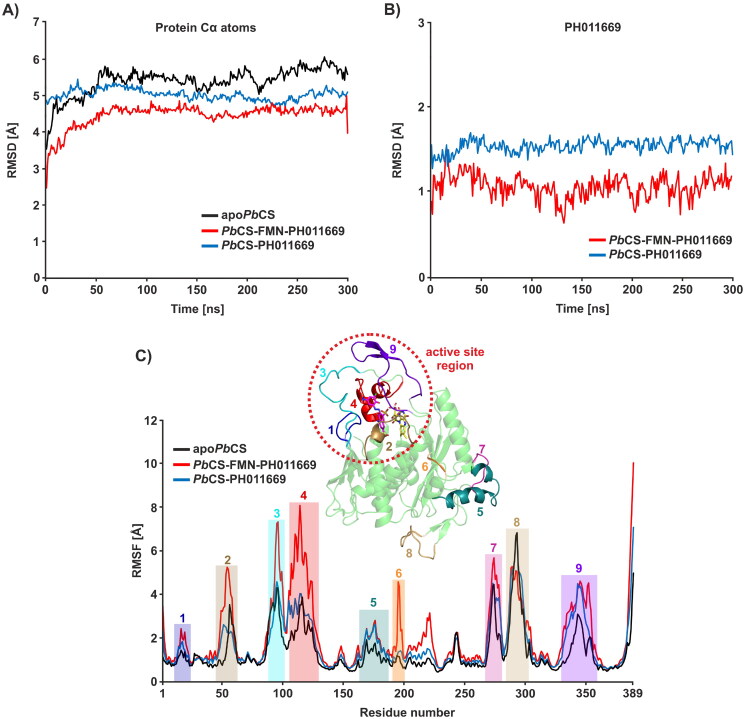
RMSD and RMSF plots for all systems. (A) RMSD plots for the Cα atoms of *Pb*CS in all systems. (B) RMSD plots for the atoms of PH011669 in the binary and ternary complex. (C) RMSF plots for all systems. Residue regions exhibiting pronounced flexibility are annotated and marked by coloured boxes. The *Pb*CS model is depicted as a green cartoon with the flexible regions annotated and coloured according to the RMSF profile to visualise where those regions are located in the structure. The dashed red circle highlights the active site region. RMSD and RMSF values were averaged over three trajectories for the apo and ternary systems and over nine trajectories for the binary system.

Comparison of the docking results of PH011669 and EPSP show that the inhibitor and substrate exhibit comparable binding modes with *Pb*CS (Figure 7C). Both molecules interact with the same set of arginine residues (i.e. R46, R103 and R362); however, in contrast to PH011669, EPSP is engaged in further salt bridges with R129 and R354 via its 3-phosphate group. Superimposition of EPSP and PH011669 show that their carboxy and sulfonate groups, respectively, are located at approximately the same positions within the binding site; the distance between the carboxy groups in EPSP is almost identical to that between the sulfonate groups in PH011669 (Figure 7C,D). Thus, the naphthalene-2,7-disulfonate moiety of PH011669 seems to mimic the 5-enolpyruvylshikimate core of EPSP within the enzyme’s binding pocket. While the whole EPSP molecule is buried in the binding pocket of *Pb*CS, only the 5-amino-4-hydroxynaphthalene-2,7-disulfonate core of PH011669 is buried, with its 3-chloro-2-hydroxy-5-nitrophenyl group protruding out of the pocket into the bulk solvent.

### Molecular dynamics simulations

We used MD simulations of 300 ns to study the interactions between PH011669 and *Pb*CS in more detail. Simulations were run in the presence and absence of FMN because, in a previous study, we observed that the inhibitory compound CP1 bound slightly more tightly to the flavin-free than to the flavin-containing enzyme^13^. Simulations were also performed for *apoPbCS* as a reference.

Root mean square deviation (RMSD) analysis showed that all systems, apo*Pb*CS, *Pb*CS-PH011669 and *Pb*CS-FMN-PH011669, reached equilibrium after approximately 50 ns and remained stable throughout the simulation (Figure 8A). In the inhibitor-bound systems, PH011669 remained stable in the binding pocket throughout the simulation; only the 3-chloro-2-hydroxy-5-nitrophenyl group protruded from the binding pocket at times. The RMSD values of the inhibitor’s atoms are higher in the flavin-lacking *Pb*CS-PH011669 system than those in the *Pb*CS-FMN-PH011669 system, indicating less specific binding of PH011669 in the binary system, which will be addressed later (Figure 8B).

**Figure 9.**
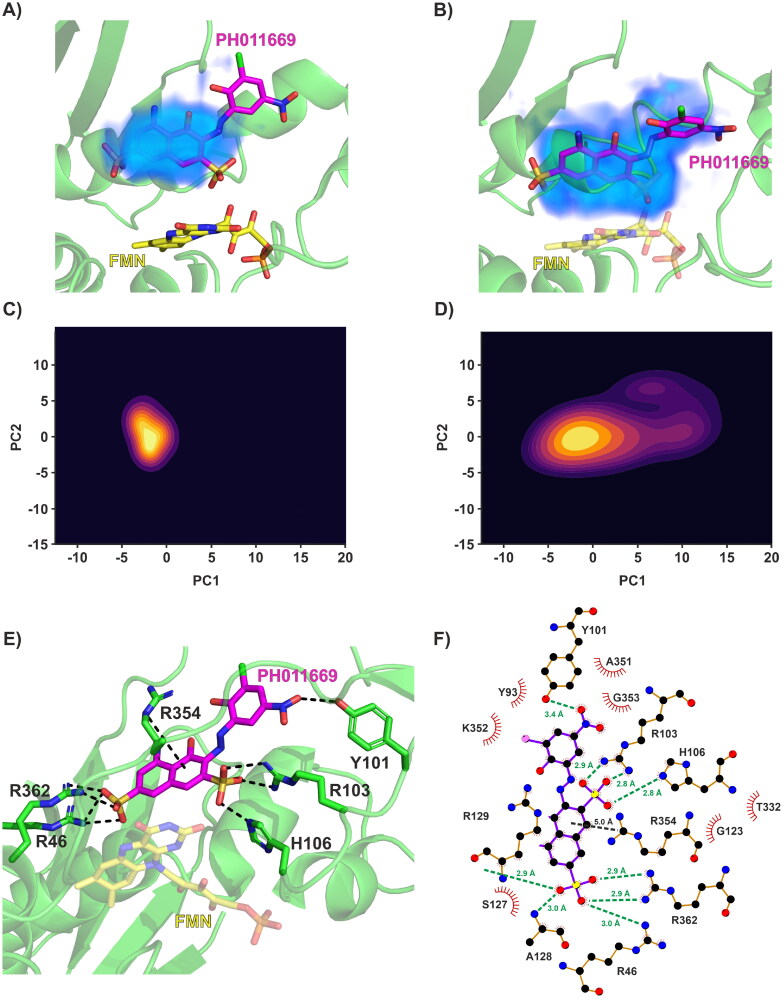
Post-molecular dynamics analysis of the *Pb*CS-FMN-PH011669 and *Pb*CS-PH011669 systems. Sampling of inhibitor conformations from the MD trajectories of the (A) ternary and (B) binary systems. Sampled conformations are shown as residence densities for PH011669 (blue volumes). For better comparison, FMN (transparent sticks) is also depicted in the FMN-lacking binary system (B). Note the differences in the size and shape of the density between the binary (B) and ternary (A) complex. Due to its high flexibility, the 3-chloro-2-hydroxy-5-nitrophenyl ring did not produce sufficient data points to be visualised as a density, so the shown densities mainly originate from the naphthalene disulfonate core. (C) Principal component analysis (PCA) of PH011669 in the ternary complex. (D) PCA of PH011669 in the binary complex. PCAs are depicted as heatmaps showing the motion of the inhibitor. The heatmaps are coloured by PH011669 density (yellow = highly populated inhibitor poses; purple = less populated inhibitor poses). (E) Representative PH011669 pose within the active site of *Pb*CS. The representative structure was taken from a frame of a trajectory located in the centre of the most populated cluster in the PCA plot (D). The enzyme is illustrated as a transparent green cartoon, with interacting residues shown as sticks. Black dashed lines indicate electrostatic and polar interactions. The interaction between R354 and the inhibitor is a cation-π interaction. (F) Protein–ligand interaction plot (LigPlot) of the representative PH011669 pose. Green dashes indicate electrostatic and polar interactions while black dashes indicate cation-π interactions; bond distances are also shown.

The root mean square fluctuation (RMSF) profiles show that the amino acid residues in the PH011669-bound systems fluctuated more than those in the apo system (Figure 8C). Besides the termini, nine regions exhibited pronounced residue flexibility (RMSF > 3 Å) in the inhibitor-bound systems, most of which were loop regions. Five of these nine flexible regions constitute the substrate binding site, which comprises the loops L1, L3, L4, L6–L8 and L20–L22, the α-helices α2–α4 and the β-sheets β11 and β12. The inhibitor-bound complexes exhibited lower RMSD but higher RMSF values than apo*Pb*CS, suggesting that PH011669 induces local structural changes within the binding region during the binding event (Figure 8A,C).

#### MD Analysis of the PbCS-FMN-PH011669 system

PH011669 exhibited only one binding mode in the ternary complex throughout the simulation. The inhibitor was stably bound within the binding site and showed no significant fluctuations, except for its 3-chloro-2-hydroxy-5-nitrophenyl moiety, which underwent considerable rotations and movements during the simulation. The 3-chloro-2-hydroxy-5-nitrophenyl group was mainly located at the entrance of the active site in direct contact with the bulk solvent and failed to engage in significant interactions with the enzyme, explaining its high flexibility for most of the simulation time.

As predicted by molecular docking, PH011669 binding is dominated by ionic interactions between the inhibitor’s negatively charged sulfonate groups and the enzyme’s positively charged amino acid residues: R46, R103, R129 and R362. These main binding contributors were within interaction distance with PH011669 (<4 Å) throughout the simulation, except for R362, which at the end of the simulation slightly lost contact with PH011669, as it shifted toward D356 to form a salt bridge (Supplementary Figure 1A–D). R46 and R362 interacted with the inhibitor’s 7-sulfonate group, whereas R103 bound to its 2-sulfonate group (Supplementary Figure 1A,B,D). Besides interacting with the 2-sulfonate group, R103 was also engaged in a moderate electrostatic interaction with the nitro group of the 3-chloro-2-hydroxy-5-nitrophenyl moiety, which, however, failed to stabilise this group notably (Supplementary Figure 1B). R129 was mainly located above the naphthalene moiety of PH011669, exhibiting cation-π interactions with the ring system for a significant fraction of the simulation time. Furthermore, it interacted moderately with the 7-sulfonate group and very weakly with the 2-hydroxy group of the 3-chloro-2-hydroxy-5-nitrophenyl moiety (Supplementary Figure 1C).

Even though the active site mainly comprises flexible regions, R46, R103, R129 and R362 did not significantly fluctuate in their positions, as evidenced by their location in the RMSF plot (Supplementary Figure 1E). Supplementary Figure 1F,G show the interactions between PH011669 and the enzyme using the energy-minimised structure of *Pb*CS-FMN-PH011669 as a representative structure, as most of these interactions persisted during the simulation.

We also determined the contact frequency (i.e. the time in percent that atoms of a given residue were within 4 Å of PH011669) and type of interaction for each residue that was interacting with PH011669 during the simulation to provide a comprehensive picture of PH011669 binding to *Pb*CS-FMN (Supplementary Table 3).

#### MD Analysis of the PbCS-PH011669 system

In contrast to the ternary *Pb*CS-FMN-PH011669 system, the *Pb*CS-PH011669 system did not exhibit a single dominant binding mode. Initially, we ran three independent MD runs for each system to ensure the robustness of the simulation, but in the binary system, the inhibitor exhibited a different binding mode in each trajectory. Therefore, we performed six more runs (in total, nine 300-ns runs) to improve the conformational sampling (and statistics) and identify a dominant binding mode for PH011669 in the flavin-free system.

After increasing the sampling, we investigated the range of motion of PH011669 during the MD simulations by calculating its residence density with the density tool of the MDAnalysis toolkit^25^^,^^26^. This tool generates a three-dimensional grid and counts the number of times an atom of the inhibitor has spent at each grid point during the simulation, providing a time-averaged distribution of the inhibitor’s atoms within a specific volume (i.e. the enzyme’s binding pocket). The analysis revealed that despite increasing the sampling, PH011669 still exhibited a markedly larger and more diffused residence density in the flavin-free than in the flavin-containing system, confirming that the inhibitor sampled a wide range of conformations and poses during the simulation of the binary system (Figure 9A,B). On the other hand, the inhibitor produced a concentrated and well-defined residence density in the ternary system, verifying the existence of only one dominant binding mode.

To better understand the dynamic behaviour of PH011669, we carried out PCA on the Cartesian coordinates of the inhibitor in both systems. The PCA plots corroborate the presence of one major binding mode for the inhibitor in the ternary system and multiple binding poses and conformations in the binary system; however, the plot of the binary system also shows a cluster of similar inhibitor conformations (Figure 9C,D). Looking at the PCA plots of the averaged trajectories (Figure 9C,D) and those of each trajectory separately (Supplementary Figure 2), it is evident that the inhibitor is relatively stable in each trajectory but not across the trajectories. Therefore, we concluded that there are several stable binding poses for PH011669 within the flavin-free system, and once the inhibitor adopts one of these poses, it remains stably bound. This could explain the previously observed slightly higher affinity of the inhibitory compound CP1 toward FMN-free *Pb*CS, as the inhibitor may have exhibited multiple stable binding conformations in the absence of FMN, outperforming the binding of the inhibitor’s primary binding mode in FMN-loaded *Pb*CS^13^.

The MD trajectories of the *Pb*CS-PH011669 system showed binding poses for the inhibitor that were similar to that in the *Pb*CS-FMN-PH011669 system (highest populated poses in the binary system) but also poses in which the inhibitor intruded deeper into the binding pocket, but only partially overlapping with the FMN binding site. PH011669 interacted mainly with the same set of arginine residues as in the ternary complex (i.e. R46, R103, R129 and R362) and was therefore electrostatically trapped above the FMN binding site for most of the simulation time. Figure 9E,F show the enzyme–inhibitor interactions of a representative structure from a trajectory that is located in the center of the major cluster in the PCA plot, thus representing the most populated binding pose for the binary system (Figure 9D). In this binding pose, PH011669 is located above the FMN binding site and interacts via ionic interactions with R46, R103, R129 and R362 and via H-bonding with Y101, H106 and the backbone of A128.

Collectively, the lack of FMN seems to lead to a less specific—but still tight—binding of PH011669 to *Pb*CS compared with that in the presence of FMN. The main contributors to PH011669 binding (i.e. R46, R103, R129 and R362) are spatially orchestrated so that ligand binding above the FMN binding site is favoured in CSs. However, our MD simulations also suggest that in the absence of FMN, PH011669 exploits the increased space and freedom of movement within the active site to a certain degree, exhibiting multiple stable binding conformations. We did not observe any significant FMN-induced structural changes that affected inhibitor binding. This is also corroborated by the fact that the active sites of experimentally determined apo structures of CSs are almost identical to that of the FMN-bound CS structure 1QXO, exhibiting Cα-RMSD values between 0.7 and 1.5 Å.

#### Binding free energy estimation and decomposition

To evaluate the affinity of PH011669 to both systems and verify our findings, we estimated the binding free energies using the MM/PBSA method. The average Gibbs binding free energies (ΔG_binding_) for the *Pb*CS-FMN-PH011669 and *Pb*CS-PH011669 systems were −50.2 kcal/mol and −39.6 kcal/mol, respectively (Table 3). MM/PBSA calculations confirmed that electrostatic interactions (ΔE_EL_) govern PH011669 binding in both systems. However, the positive solvation-free energy change (ΔG_SOL_) and the negative gas-phase contribution to the free energy change (ΔG_GAS_) indicate strong interactions between PH011669 and the solvent. This is corroborated by trajectory structures in which the 3-chloro-2-hydroxy-5-nitrophenyl group of the inhibitor was highly solvent-exposed in both systems. Thus, our Gibbs binding free energy analysis suggests that reducing the polarity and size of the inhibitor should be considered to improve its binding to *Pb*CS.

**Table 3.** Gibbs free energies calculated by MM/PBSA.

	Energies [kcal/mol]	
	*Pb*CS-FMN-PH011669	*Pb*CS-PH011669
ΔE_VDW_	−55.35 ± 0.07	−47.11 ± 0.06
ΔE_EL_	−123.52 ± 0.41	−154.79 ± 0.37
ΔE_POL_	135.78 ± 0.34	206.78 ± 0.33
ΔE_NONPOL_	−7.05 ± 0.01	−6.48 ± 0.01
ΔG_GAS_	−178.86 ± 0.40	−239.90 ± 0.37
ΔG_SOLV_	128.73 ± 0.34	200.3 ± 0.32
ΔG_binding_	−50.20 ± 0.11	−39.59 ± 0.10

ΔE_VDW_: van der Waals energy; ΔE_EL_: electrostatic contribution to the solvation energy; ΔE_POL_: polar component of the solvation energy; ΔE_NONPOL_: non-polar component of the solvation energy; ΔG_GAS_: total gas phase free energy; ΔG_SOLV_: total solvation free energy; ΔG_binding_: binding free energy. The values are averages over all trajectories (three for the ternary system and nine for the binary system).

**Figure 10.**
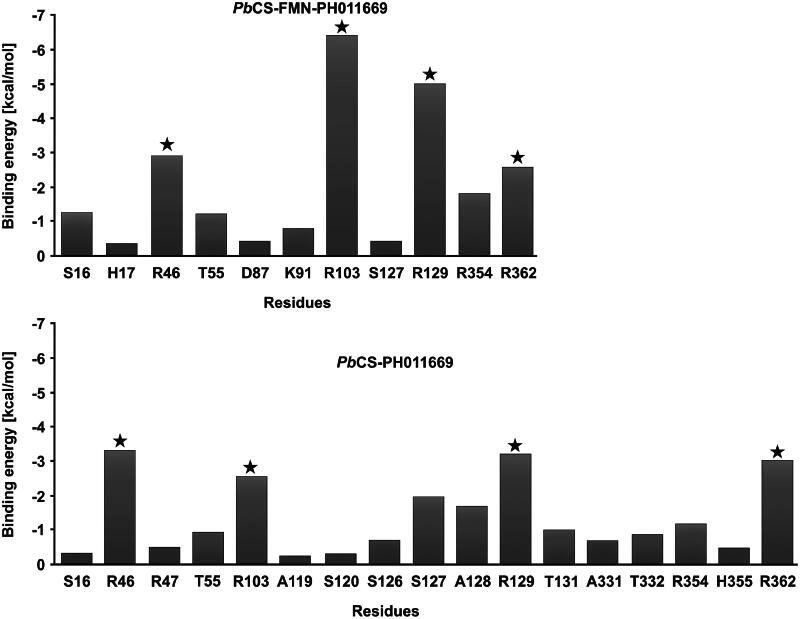
Per-residue Gibbs binding free energy decomposition analysis of the *Pb*CS-FMN-PH011669 (top panel) and *Pb*CS-PH011669 system (bottom panel). Stars highlight the main contributors to PH011669 binding. Values are averaged over all trajectories (three for the ternary system and nine for the binary system).

To confirm and identify the key residues for PH011669 binding, we performed a per-residue decomposition analysis and determined the contribution of all inhibitor-binding residues to ΔG_binding_. In both systems, R46, R103, R129 and R362 contributed the most to ΔG_binding_ (Figure 10). However, the total binding energy of PH011669 was distributed more evenly over more residues in the binary than in the ternary system, corroborating the less specific binding of PH011669 in the binary complex.

### ADME properties of PH011669 and optimisation strategies

To evaluate the propensity of PH011669 as a drug, we analysed the ADME properties of the inhibitor (Table 4). As a large molecule decorated with many functional groups, PH011669 violates two of the four Lipinski’s rule of 5, as it has a molecular mass of > 500 Da (518.9 Da) and >10 H-bond acceptors^31^. Furthermore, PH011669 exhibits a large topological polar surface area of 262.5 Å^2^, which most likely will reduce its bioavailability significantly. The presence of an azo-group is also undesirable because of the instability towards reduction, releasing potentially harmful aromatic amines^32^. Therefore, PH011669 must be optimised to achieve an acceptable pharmacokinetic profile. Our *in-silico* results indicate that PH011669 binding mainly relies on ionic interactions of its 5-amino-4-hydroxynaphthalene-2,7-disulfonate core, while the 3-chloro-2-hydroxy-5-nitrophenyl moiety contributes only marginally to the binding. In addition, the binding contributions of the 5-amino and 4-hydroxy groups seem also negligible. Thus, removing these groups is expected to hardly affect the inhibitor’s binding but improve its pharmacokinetic profile significantly. To also enhance the binding affinity of PH011669, we propose adding a negatively charged group, such as a carboxy group, instead of the 4-hydroxy group to enable further salt bridge formation with R129 and R354, as observed for EPSP binding in *Sp*CS (Figure 7C,D). Applying these modifications would lead to a pharmacologically more suitable inhibitor, as evidenced by a lower molecular mass of 332.3 Da, the absence of the azo group and a lower topological polar surface area of 162.8 Å^2^, which is still considered high but very close to the upper limit of the Muegge filter^33^ (Table 4).

**Table 4.** Lipinski’s rule of 5 violations and ADME properties of PH011669 and its optimised version.

	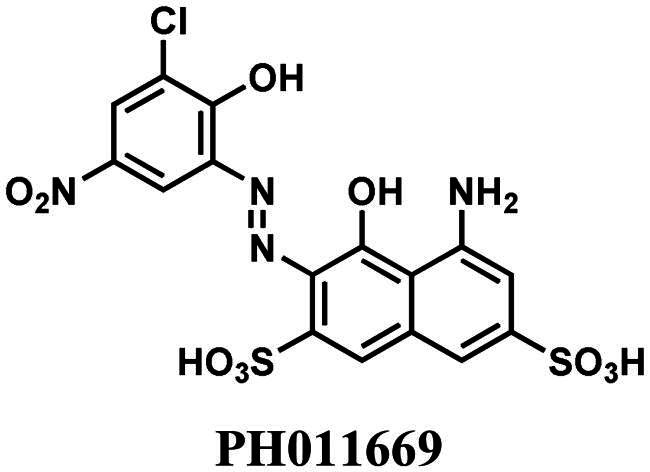	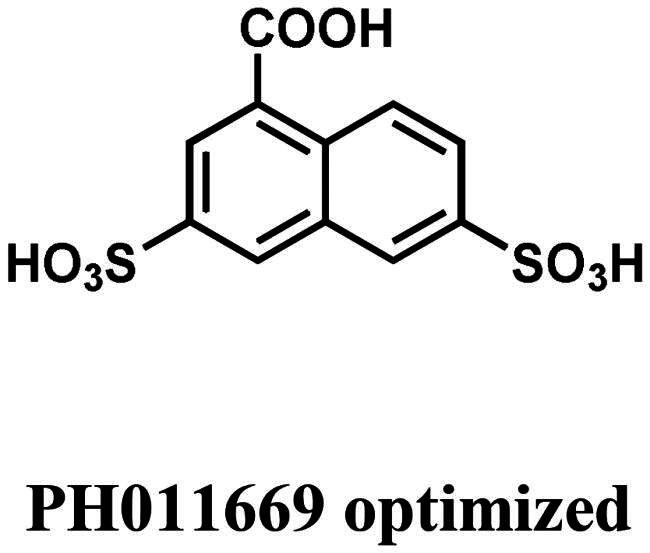
Lipinski’s rule of 5		
150 Da ≤ MW ≤500 Da	518.9 Da	332.3 Da
−0.7 ≤ Log P ≤ 5	2.06	0.19
≤10 H-bond acceptors	12	8
≤5 H-bond donors	5	3
		
Other pharmacokinetic properties		
20 Å^2^ ≤ TPSA ≤150 Å^2^	262.5 Å^2^	162.8 Å^2^
−6 ≤ Log S ≤ 0	−4.38	−2.17
No. of rotatable bonds ≤ 7^a^	5	3
Presence of azo group	Yes	No
Gastrointestinal absorption^b^	Low	Low
Interactions with CYP isoenzymes^c^	No	No

MW: molecular weight; TPSA: topological polar surface area; CYP: cytochromes P450; BBB: blood-brain barrier.

^a^Drugs with more than seven rotatable bonds are flexible and can lead to unfavourable ADME properties, as the increased conformational entropy can distort target binding.

^b^For a drug to be effective, its gastrointestinal absorption should be high to enter the bloodstream.

^c^Assessing the inhibitory interactions of a drug with the CYP isoenzymes CYP1A2, CYP2C19, CYP2C9, CYP2D6 and CYP3A4 is essential because inhibition of these isoenzymes can lead to drug–drug interactions, increasing the risk of adverse effects. In this category, “no” means that the drug is not an inhibitor of any CYP isoenzyme.

## Conclusions

The shikimate pathway is essential for prokaryotes, protozoa, fungi and plants but is absent in humans, rendering the enzymes of this pathway, such as CS, attractive targets for developing antifungal or antimicrobial agents. In this study, we screened commercially available azo-dyes for CS inhibitors and identified the novel lead compound PH011669 for the development of highly potent therapeutic agents targeting the shikimate pathway. Molecular docking and dynamics simulations predicted that the azo-dye binds to the active site of *Pb*CS primarily via strong ionic interactions between its negatively charged sulphonate groups and three arginine residues (R46, 103 and R362). These interactions essentially mimic those observed in the crystal structure of *Sp*CS in complex with its natural substrate EPSP. MD simulations in the presence and absence of the FMN cofactor demonstrated that the interacting arginine residues R46, R103 and R362 are arranged in such a way within the binding site so that they can orchestrate the binding of negatively charged ligands directly above the cofactor. The calculated binding poses showed that the naphthalene-2,7-disulfonate core of PH011669 is mainly responsible for its binding to CS, suggesting that the remaining functional groups can be removed without considerably affecting its binding affinity. Such modifications will be necessary to obtain a pharmacologically acceptable inhibitor that can be considered for medicinal purposes, as the current structure of PH011669 has a poor pharmacokinetic profile. Furthermore, our computational results provide valuable guidance for future inhibitor designs, as they show which hot spots in the active site of CS need to be targeted (e.g. R46, R103 and R362). For example, our results suggest that adding a further negative charge at the 4-position of the naphthalene ring of PH011669 could produce a compound with higher affinity to CS, as additional electrostatic interactions will be established.

Additional studies need to be conducted to optimise the present lead compound, especially in terms of improving its drug-likeliness, although our data can support future research toward antifungal or antimicrobial therapeutics that target the shikimate pathway via CS inhibition.

## Supplementary Material

Supplemental Material

## Data Availability

The datasets (MD trajectories) presented in this study have been deposited in the repository at TU Graz (https://repository.tugraz.at/records/p37ee-ph630).
