# Supplementary material for: Investigation of the inhibitory properties of azo-dyes on chorismate synthase from Paracoccidioides brasiliensis
Source: J Enzyme Inhib Med Chem. 2024 Dec 10;39(1):2427175. doi: 10.1080/14756366.2024.2427175 (PMC11633415; doi:10.1080/14756366.2024.2427175)
Supplement: Supplemental Material [file IENZ_A_2427175_SM0217.docx]

**Investigation of the inhibitory properties of azo-dyes on chorismate synthase from *Paracoccidioides brasiliensis***

Katharina Fuchs^a#^, Massimo G. Totaro^a#^, Marina Toplak^a^, Aleksandar Bijelic^a^ and Peter Macheroux^a*^

*^a^Graz University of Technology, Institute of Biochemistry, Graz, Austria*

^#^ These authors contributed equally

* To whom correspondence should be addressed:

Prof. Dr. Peter Macheroux, Institute of Biochemistry, Graz University of Technology, Petersgasse 12/II, A-8010 Graz, Austria; Email: peter.macheroux@tugraz.at

# Supplementary Tables

**Supplementary Table 1.** Azo-dyes investigated in the current study.

| **Inhibitor^a^** | **IUPAC name** | **Structural formula** |
| --- | --- | --- |
| PH011669 | 5-amino-3-[(E)-(3-chloro-2-hydroxy-5-nitrophenyl) diazenyl]-4-hydroxy-2,7-naphthalenedisulfonic acid |  |
| S981796 | 3-(4-(diethylamino)phenylazo)-4,5-  dihydroxy -2,7-naphthalene disulfonic acid |  |
| 200182 | 2-(2,4-dinitrophenylazo)-1-hydroxynaphthalene-3,6-disulfonic acid  (nitrazine yellow)^b^ |  |
| S469734 | 2,7-naphthalenedisulfonic acid, 5- amino-4-hydroxy-3-[(E)-2-phenyldiazenyl]  (acid red 33 (C.I. 17200))^b^ |  |
| R324272 | 1-naphthalenesulfonic acid, 4-hydroxy-3-[(E)-2-(2-methoxyphenyl)diazenyl]  (acid red 4)^b^ |  |
| S587664 | 4-hydroxy-3-(phenylazo)-1-  naphthalenesulfonic acid |  |
| S984191 | 5-amino-4-hydroxy-3-(4-hydroxy-3-methylphenylazo)-2,7-naphthalene-disulfonic acid |  |
| S9256 | 1,8-dihydroxy-2-(4-sulfophenylazo)naphthalene-3,6-disulfonic acid  (sulfanilic acid azochromotrop)^b^ |  |
| S480762 | 3-(1,5-dihydroxy-naphthalene-2- ylazo)-4-hydroxy-benzenesulfonic acid |  |
| C3143 | 2-(phenylazo) chromotropic acid  (chromotrop 2R)^b^ |  |
| 27130 | 2-(4-nitrophenylazo) chromotropic acid  (chromotrop 2B)^b^ |  |
| S602736 | 3-(1,5-dihydroxy-2-naphthylazo)-4-hydroxybenzenesulfonic acid sodium salt |  |
| S285668 | disodium-4,5-dihydroxy-3-[(2-methyl-4-sulfonatophenyl)diazenyl]-1-naphthalenesulfonate |  |
| S480835 | disodium (3E)-3-[(4-acetamidophenyl)hydrazono]-4-oxo-3,4-dihydro-2,7-naphthalenedisulfonate  (acid red 7 (C.I. 14895))^b^ |  |
| 306509 | 2-naphthalenesulfonic acid, 6-(acetylamino)-4-hydroxy-3-[(E)-2-[4-[[2-(sulfooxy)ethyl]sulfonyl]phenyl] diazenyl  (reactive orange 16)^b^ |  |

^a^Sigma-Aldrich product number

^b^Trivial name

**Supplementary Table 2.** Expression vectors and strains used to produce the CSs from different organisms.

| **Organism** | ***E. coli* expression vector** | ***E. coli* expression strain** |
| --- | --- | --- |
| *Aquifex aeolicus* | pET-28c(+) | BL21 (DE3) RIL |
| *Anabaena variabilis* | pET-21a(+) | BL21 (DE3) RIL |
| *Botrytis fuckeliana* | pET-28a(+) | BL21Star (DE3) |
| *Candida albicans* | pET-21a(+) | BL21Star (DE3) |
| *Corydalis sempervirens* | pET-21a(+) | BL21Star (DE3) |
| *Escherichia coli* | pET-21d(+) | BL21Star (DE3) |
| *Methanobacterium thermoautotrophicum* | pET-28c(+) | BL21 (DE3) RIL |
| *Mycobacterium tuberculosis* | pET-23a(+) | Rosetta (DE3) |
| *Neurospora crassa* | pET-21a(+) | BL21 (DE3) RP |
| *Paracoccidioides brasiliensis* | pET-21a(+) | BL21 (DE3) |
| *Staphylococcus aureus* | pET-21a(+) | BL21 (DE3) RIL |
| *Saccharomyces cerevisiae* | pET-21d(+) | BL21 (DE3) RIL |
| *Solanum lycopersicum* | pET-21a(+) | BL21 (DE3) RIL |
| *Toxoplasma gondii* | pET-21a(+) | BL21 (DE3) RIL |
| *Thermotoga maritima* | pET-28c(+) | BL21 (DE3) RIL |
| *Tetrahymena thermophila* | pET-28c(+) | BL21 (DE3) RIL |

**Supplementary Table 3.** Per-residue contact frequencies between *Pb*CS-FMN and PH011669.

| ***Pb*CS residue^a^** | **Interaction type** | **Contact frequency [%]^b^** |
| --- | --- | --- |
| S16 | H-bond | 41 |
| R46 | electrostatic/salt bridge | 95 |
| R103 | electrostatic/salt bridge | 99 |
| H106 | H-bond | 40 |
| S120 | polar (backbone) + H-bond | 42 |
| S127 | H-bond | 72 |
| A128 | H-bond (backbone) | 69 |
| R129 | H-bond + cation-π | 100 |
| I132 | apolar | 45 |
| R354 | electrostatic/salt bridge + cation-π | 36^c^ |
| R362 | electrostatic/salt bridge | 70 |
| D87 | H-bond | 69 |

^a^ Only residues with a contact frequency of at least 40% are shown; a contact was counted when at least one atom of a residue was within 4 Å of PH011669.
^b^ Contact frequencies are given as a percentage of all sampled frames.
^c^ Despite having a contact frequency below 40%, R354 is listed because it contributes to the binding energy (see Figure 10 in the main text).

# Supplementary Figures

Supplementary Figure 1

**
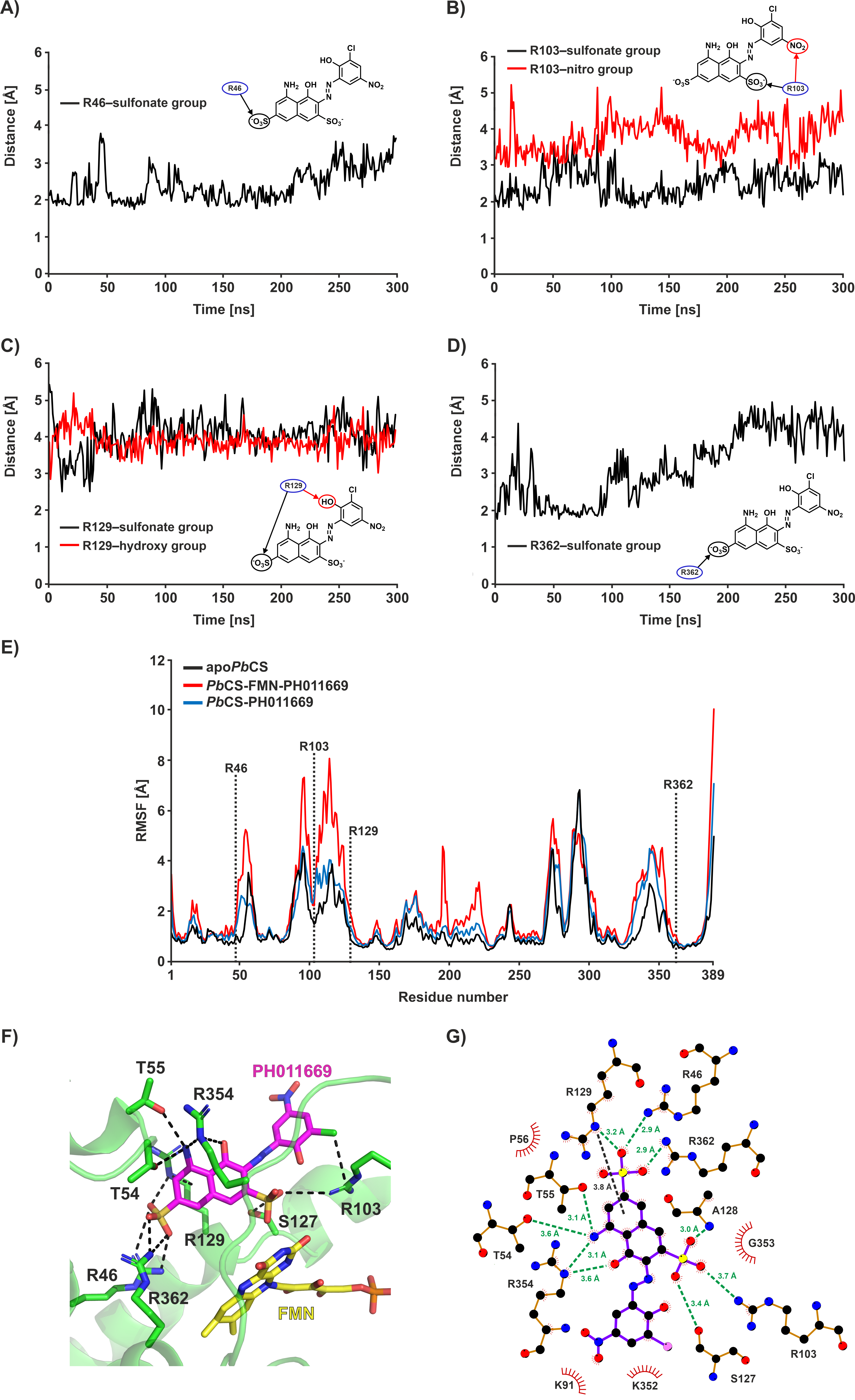
**

**Supplementary Figure 1.** Breakdown of the interactions in the *Pb*CS-FMN-PH011669 system. (A) – (D) Interaction distances (averaged over three trajectories) between the functional groups of PH011669 and R46, R103, R129, and R362 plotted over simulation time. Insets illustrate the plotted interactions for clarity. (E) RMSF plots of all systems, with the positions of the main PH011669-binder highlighted. Despite being located on flexible regions (RMSF >3 Å), the residues themselves exhibited relatively low RMSF values (RMSF <2 Å). (F) Energy-minimized binding pose of PH011669 within the active site of *Pb*CS-FMN. The enzyme is illustrated as a transparent green cartoon, with interacting residues shown as sticks. Black dashed lines indicate electrostatic and polar interactions. The interaction between R129 and the inhibitor is a cation-π interaction. (G) Protein–ligand interaction plot (LigPlot) of the energy-minimized *Pb*CS-FMN-PH011669 complex. Green dashes indicate electrostatic and polar interactions while black dashes indicate cation-π interactions.

**
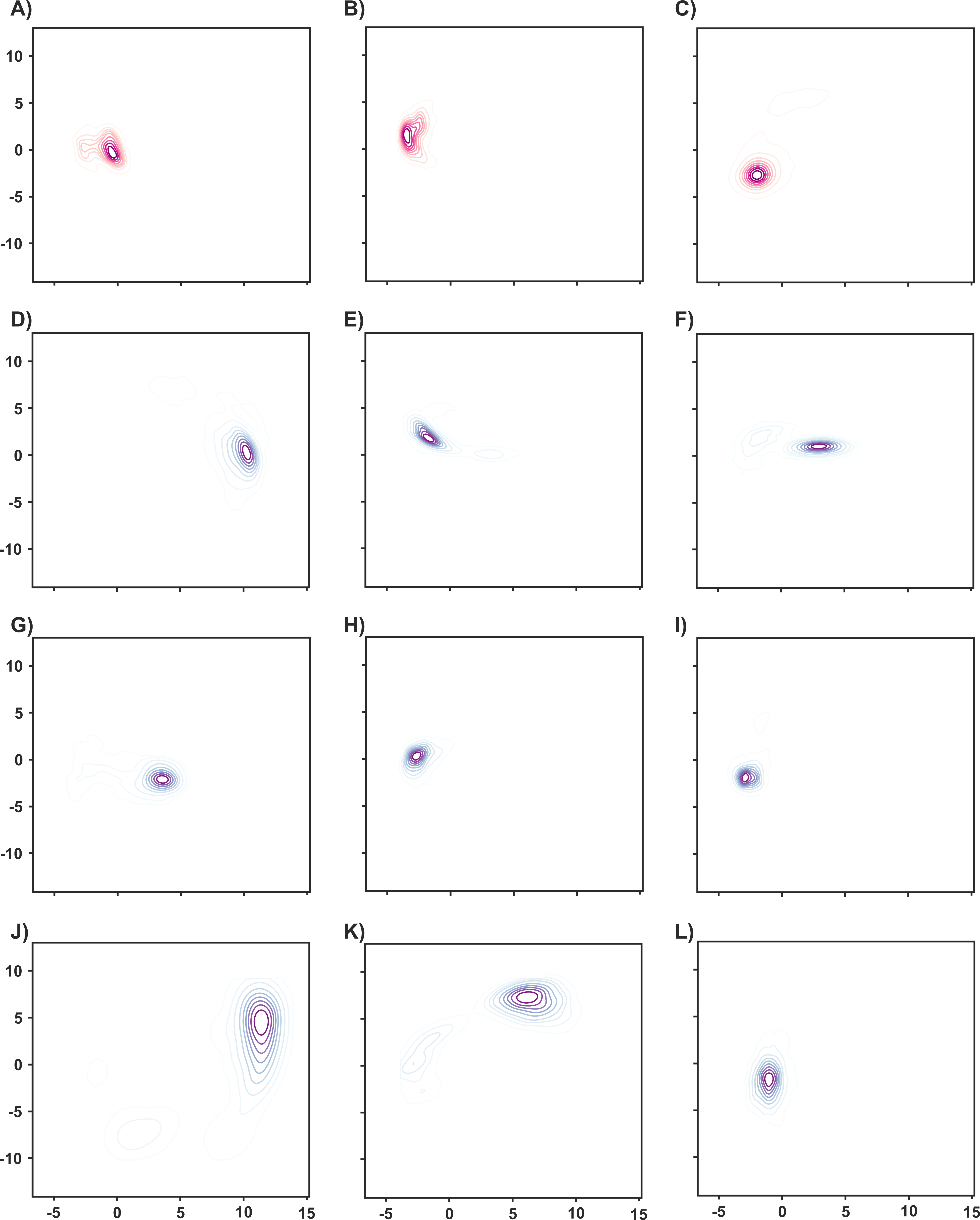
**

**Supplementary Figure 2.** Principal component analysis (PCA) for each trajectory (binary and ternary system). Shown are the PH011669 density distributions of the MD frames plotted against the first two PCA vectors. (A) – (B) PCA for the three independent trajectories of the ternary system. (C) – (L) PCA for the nine independent trajectories of the binary system.
